# Are people who have a better smell sense, more affected from satiation?^☆^

**DOI:** 10.1016/j.bjorl.2016.08.011

**Published:** 2016-09-12

**Authors:** Seckin Ulusoy, Mehmet Emre Dinc, Abdullah Dalgic, Murat Topak, Denizhan Dizdar, Abdulhalim İs

**Affiliations:** a Turkish Ministry of Health, Gaziosmanpasa Taksim Education and Research Hospital, Department of Otorhinolaryngology, Istanbul, Turkey; b İstanbul Kemerburgaz University, Faculty of Medicine, Bahçelievler Medical Park Hospital, İstanbul, Turkey

**Keywords:** Sniffin’ Sticks test, Fasting period, Satiated period, Humans, Smell function, Teste de *Sniffin’ Sticks*, Período de jejum, Período de saciedade, Seres humanos, Função do olfato

## Abstract

**Introduction:**

The olfactory system is affected by the nutritional balance and chemical state of the body, serving as an internal sensor. All bodily functions are affected by energy loss, including olfaction; hunger can alter odour perception.

**Objective:**

In this study, we investigated the effect of fasting on olfactory perception in humans, and also assessed perceptual changes during satiation.

**Methods:**

The “Sniffin’ Sticks” olfactory test was applied after 16 h of fasting, and again at least 1 h after Ramadan supper during periods of satiation. All participants were informed about the study procedure and provided informed consent. The study protocol was approved by the local Ethics Committee of Gaziosmanpaşa Taksim Education and Research Hospital (09/07/2014 no: 60). The study was conducted in accordance with the basic principles of the Declaration of Helsinki.

**Results:**

This prospective study included 48 subjects (20 males, 28 females) with a mean age of 33.6 ± 9.7 (range 20–72) years; their mean height was 169.1 ± 7.6 (range 150.0–185.0) cm, mean weight was 71.2 ± 17.6 (range 50.0–85.0) kg, and average BMI was 24.8 ± 5.3 (range 19.5–55.9). Scores were higher on all items pertaining to olfactory identification, thresholds and discrimination during fasting vs. satiation (*p* < 0.05). Identification (I) results: Identification scores were significantly higher during the fasting (median = 14.0) vs. satiation period (median = 13.0). Threshold (T) results: Threshold scores were significantly higher during the fasting (median = 7.3) vs. satiation period (median = 6.2). Discrimination (D) results: Discrimination scores were significantly higher during the fasting (median = 14.0) vs. satiation period (median = 13.0). The total TDI scores were 35.2 (fasting) vs. 32.6 (satiation). When we compared fasting threshold value of >9 and ≤9, the gap between the fasting and satiety thresholds was significantly greater in >9 (*p* < 0.05).

**Conclusion:**

Olfactory function improved during fasting and declined during satiation. The olfactory system is more sensitive, and more reactive to odours, under starvation conditions, and is characterised by reduced activity during satiation. This situation was more pronounced in patients with a better sense of smell. Olfaction-related neurotransmitters should be the target of further study.

## Resumo

**Introdução:**

O sistema olfatório é afetado pelo equilíbrio nutricional e estado químico do corpo, que serve como um sensor interno. Todas as funções corporais são afetadas pela perda de energia, incluindo o olfato; a fome pode alterar a percepção do odor.

**Objetivo:**

Neste estudo, investigamos o efeito do jejum sobre a percepção olfativa em seres humanos, e também avaliamos as mudanças de percepção durante a saciedade.

**Método:**

O teste olfatório *Sniffin Sticks* foi aplicado após 16 horas de jejum e novamente pelo menos 1 hora após a ceia do Ramadã durante os períodos de saciedade. Todos os participantes foram informados sobre os procedimentos do estudo e forneceram o consentimento informado. O protocolo do estudo foi aprovado pelo Comitê de Ética local do Gaziosmanpaşa Taksim Education e Research Hospital (2014/09/07 n° 60). O estudo foi conduzido de acordo com os princípios básicos da Declaração de Helsinki.

**Resultados:**

Foram incluídos 48 pacientes (20 homens, 28 mulheres) com idade média de 33,6 ± 9,7 (variação 20-72) anos; a altura média deles era de 169,1 ± 7,6 (variação 150,0-185,0) cm, o peso médio era de 71,2 ± 17,6 (variação de 50,0-85,0) kg e IMC médio era de 24,8 ± 5,3 (variação de 19,5-55,9). Os escores foram maiores em todos os itens correspondentes à identificação olfativa, limiares e discriminação durante jejum vs. saciedade (*p* < 0,05). Resultados da identificação (I): os escores de identificação foram significativamente maiores durante o jejum (mediana = 14,0) vs. período de saciedade (mediana = 13,0). Resultados limiares (T): os escores limiares foram significativamente maiores durante o jejum (mediana = 7,3) vs. período de saciedade (mediana = 6,2). Resultados de discriminação (D): os escores de discriminação foram significativamente maiores durante o jejum (mediana = 14,0) vs. período de saciedade (mediana = 13,0). Os escores totais de TDI foram de 35,2 (jejum) vs. 32,6 (saciedade). Quando comparamos o valor do limiar de jejum de >9 e ≤9, a diferença entre os limiares de jejum e de saciedade foi significativamente maior em >9 (*p* < 0,05)

**Conclusão:**

A função olfatória melhorou durante o jejum e diminuiu durante a saciedade. O sistema olfatório é mais sensível e mais reativo aos odores em condições de fome e é caracterizado por atividade reduzida durante a saciedade. Esta situação foi mais pronunciada em pacientes com um melhor sentido olfativo. Os neurotransmissores relacionados com o olfato devem ser alvo de um estudo mais aprofundado.

## Introduction

All bodily functions are affected by energy loss, including olfaction; hunger can alter odour perception. Changes in subjective evaluation of an unchanging food stimulus are commensurate with changes in hunger state^1^; recent evidence suggests that hunger state can similarly affect food odour pleasantness.^2^ Although the mechanisms underlying alterations for food and odour stimuli (e.g., from positive to negative following satiation) are not yet understood, loss of energy is linked to changes in olfactory bulb activity^3^ and olfactory sensitivity in rats.^1^, ^4^

The olfactory system is affected by the nutritional balance and chemical state of the body, serving as an internal sensor.^1^ The endocrine and olfactory systems are linked closely. Hormones and metabolic peptides may be orexigenic or anorexigenic, depending on their inhibition or stimulation of food intake. The hypothalamus and several other parts of the brain, including olfactory regions, are stimulated by leptin and insulin, causing an anorexigenic effect.^5^

Farhadian et al.^6^ studied the relationship between post-fasting behaviour and changes in olfactory responsiveness, and suggested that the olfactory system is affected by nutritional status: fasted flies were more receptive to attractive odours compared with satiated flies. This phenomenon was demonstrated in the nematode *Caenorhabditis elegans*. Worms typically react to the smell of octanol by moving backwards, but in the absence of food this response is significantly less rapid.^7^

In this study, we investigated the effect of fasting on olfactory perception in humans, and also assessed perceptual changes during satiation. The “Sniffin’ Sticks” olfactory test was administered during Ramadan fasting and during subsequent periods of satiation. Identification, threshold and discrimination scores were evaluated. Scores for all of the test items pertaining to these three categories were significantly higher during fasting than during satiation.

## Methods

Forty-eight subjects (20 males, 28 females) admitted to the Ear, Nose and Throat (ENT) Clinic of the Gaziosmanpaşa Taksim Education and Research Hospital between June 28, 2014 and August 27, 2014 were enrolled. All patients were participating in Ramadan fasting. The “Sniffin’ Sticks” olfactory test was applied after 16 h of fasting, and again at least 1 h after Ramadan supper during a period of satiation. The mean age of patients was 33.5 ± 9.6 years.

All participants were informed about the study procedure and provided informed consent. The study protocol was approved by the local Ethics Committee of the Gaziosmanpaşa Taksim Education and Research Hospital (09/07/2014 n° 60). The study was conducted in accordance with the basic principles of the Declaration of Helsinki.

### Patient selection

The following inclusion criteria were applied: (1) participating in Ramadan fasting; (2) no pre-existing medical, surgical or psychiatric comorbid conditions; (3) no physical or psychological disabilities that would affect participation; (4) no history of medication use except daily supplemental vitamins and iron pills; (5) no previous diagnosis of upper airway disease nor previous nasal surgery; and (6) in the first period of the menstrual cycle (females only). Smokers and menopausal females were excluded. All subjects underwent an ENT examination, conducted by ENT specialists, to confirm the absence of upper airway disease.

### Evaluation of olfactory function

“Sniffin’ Sticks” olfactory tests (Burghart, Wedel, Germany)^8^, ^9^ – i.e., pen-like odour dispensing devices – were used to assess olfaction. Odour threshold, discrimination, and identification parameters were measured. To present each odour, caps were removed from the sticks by the researcher, with the tip then held approximately 2 cm in front of both nostrils of the participant for approximately 3 s. Subjects were blindfolded to prevent visual identification of the odour-containing pens. For threshold testing, each pen's tampon was filled with phenyl ethyl alcohol (PEA; characterised by a rose-like odour) diluted in propylene glycol (dilution ratio = 1:2, starting at 4%). PEA odour threshold was assessed using a single-staircase, three-alternative forced-choice (3-AFC) procedure. Three pens were presented to each subject randomly; two contained an odourless solvent (propylene glycol), and the third contained an odourant of a certain dilution. Three new pens were presented at 20 s intervals, and the subject was required to indicate the pen containing the odourant. The concentration of the odour-containing pen was increased if the subject selected one of the odourless pens, and decreased if the odourant was selected. The mean of the previous four, of seven total, reversal points was accepted as the detection threshold (range 1–16).^10^ For odour discrimination, 16 sets of three pens were presented, two of which contained identical odourants; the third contained the target odourant. Subjects were asked to identify the unique sample; the number of correctly identified odours was summed to produce the test score. Odour identification was assessed using 16 common odours and a multiple forced-choice design; subjects identified odours by selecting the most-appropriate of four different descriptions.

### Statistical analysis

Analyses were performed using the SPSS for Windows software package (ver. 22.0; SPSS, Chicago, IL, USA). According to the Kolmogorov–Smirnov test results, when the *p*-value is less than 0.05, variables are not distributed normally. Therefore, nonparametric statistical methods were used in the study. In the first stage of basic statistical data analysis, the median and range values are given. In the second stage involving the testing of group differences, Wilcoxon and McNemar tests, the latter being two-sided, were used.

## Results

This prospective study included 48 subjects (20 males, 28 females) with a mean age of 33.6 ± 9.7 (range 20–72) years; their mean height was 169.1 ± 7.6 (range 150.0–185.0) cm, mean weight was 71.2 ± 17.6 (range 50.0–85.0) kg, and average BMI was 24.8 ± 5.3 (range 19.5–55.9). The baseline characteristics of the subjects are summarised in Table 1.

**Table 1:** Baseline characteristics of the study subjects.

	Min–Max	Median	Mean ± SD/*n* (%)
*Age*	20–72	32	33.6 ± 9.7

*Gender*			
Female			28 (58%)
Male			20 (42%)

*Length*	150–185	170	169.1 ± 7.6
*Weight*	50–175	70	71.2 ± 17.6
*BMI*	19.5–55.9	23.9	24.8 ± 5.3

**Figure 1:**
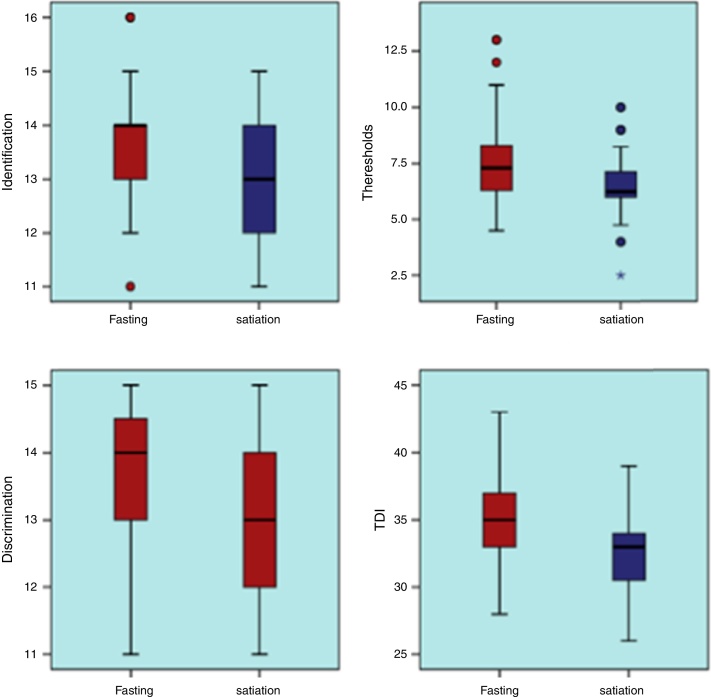
Overall olfactory function in hunger and in satiety.

Fasting and satiation period test results are displayed in Table 2 and Fig. 1.

**Table 2:** Overall olfactory function in fasting and satiation.

	Fasting		Satiation		*p*
	Mean ± SD/*n* (%)	Med (min–max)	Mean ± SD/*n* (%)	Med (min–max)	
Identification	13.7 ± 1.1	14 (11–16)	12.8 ± 1.1	13 (11–15)	0.000
Thresholds	7.7 ± 1.9	7.3 (4.5–13)	6.5 ± 1.4	6.3 (2.5–10)	0.000
≤9	39 (81%)		46 (96%)		0.008
>9	9 (19%)		2 (4%)		
Discrimination	13.8 ± 1.0	14 (11–15)	13.2 ± 1.1	13 (11–15)	0.000
TDI	35.2 (3.5)	35 (28–43)	32.6 (3.0)	33 (26–39)	0.000

Wilcoxon test/MC Nemar test.

Identification (I) results: Identification scores were significantly higher during the fasting (median = 14.0) vs. satiation period (median = 13.0).

Threshold (T) results: Threshold scores were significantly higher during the fasting (median = 7.3) vs. satiation period (median = 6.2).

Discrimination (D) results: Discrimination scores were significantly higher during the fasting (median = 14.0) vs. satiation period (median = 13.0).

The total TDI scores were 35.2 (fasting) vs. 32.6 (satiation).

A fasting threshold value of >9 was used to define Group A; the fasting period threshold value was significantly higher compared to the satiety period (*p* < 0.05).

A fasting threshold value of ≤9 was used to define Group B; the fasting period threshold value was significantly higher compared to the satiety period (*p* < 0.05) (Table 3).

**Table 3:** Compare fasting threshold value of ≤9 and >9 with satiation.

	Fasting thresholds ≤9		Satiation thresholds >9		*p*
	Mean ± SD/*n* (%)	Med (min–max)	Mean ± SD/*n* (%)	Med (min–max)	
Fasting	7.1 ± 1.1	7.0 (4.5–9.0)	10.5 ± 2.1	0.5 (6.3–13.0)	
Satiation	6.1 ± 1.1	6.3 (2.5–9.0)	8.2 ± 1.4	8.3 (6.0–10.0)	
Difference	0.9 ± 0.8	1.0 (−1.0 to 3.5)	2.3 ± 1.0	3.0 (0.3–3.3)	0.001
*p*	0.000		0.007		

Wilcoxon test/Mann–Whitney *U* test.

When we compared Groups A and B, the gap between the fasting and satiety thresholds was significantly greater in Group A (*p* < 0.05).

## Discussion

In mammals, the sense of smell is modulated by the status of satiety, which is mainly signalled by blood-circulating peptide hormones. However, the underlying mechanisms linking olfaction and food intake are poorly understood. Olfaction is a major factor in the decision to eat a food item or refuse it. Appetite-stimulating and appetite-suppressing hormones also have effects on olfactory-driven behaviour.

Orexigenic molecules (stimulatory) include ghrelin, neuropeptide Y, orexins, endocannabinoids,^11^ endogenous opioids.^11^ Anorexigenic molecules (inhibitory) include insulin,^12^ leptin,^13^ cholecystokinin,^14^ and nutrient glucose^7^ have been studied by numerous authors. When hungry or satiated, the stomach, intestines, pancreas and other organs regulate various peripheral molecules.^14^ The olfactory mucosa and bulb, as well as the hypothalamus, are targeted through blood containing these molecules. In response, metabolic factors are released by the hypothalamus to control nutritional homeostasis. The olfactory system is also affected by these changes, adapting to the nutritional needs of the body.

Serotonin may mediate hunger signals, because its administration precipitates feeding in olfactory behaviour trials^7^; furthermore, in flies antennal lobe projection neurons are enhanced by serotonin under certain conditions.^15^ Serotonin, or a similar, secreted molecule, might also regulate 3-methyl-thio-1-propanol sensitivity in flies post-starvation.

The modulation of olfactory performance has been studied in metabolic disorders such as obesity, diabetes, and anorexia nervosa. Changing levels of olfactory-modifying molecules alter brain activation and the response to food odours. Metabolic disorders disrupt olfactory performance, thereby disrupting energy balance.^16^ Changes in hormone and glucose levels are detected by receptors and peptides related to feeding. The hypothalamus and olfactory system communicate through the olfactory bulb, and caloric intake and metabolism speed are influenced by the olfactory system.^16^

Aime et al.^4^ suggested that olfaction plays a fundamental role in feeding behaviour. The relationship between olfactory acuity and feeding status has not been determined precisely in animal models; however, these authors evaluated olfactory detection in fasted and satiated rats placed under a rigorously controlled food-intake regimen, and obtained original data verifying the hypothesis that olfactory sensitivity is increased in fasted animals. Since their results were obtained using a neutral odour, the authors suggest that olfactory acuity increases that occur during fasting enable animals to more-easily detect salient environmental odours, including food items and predators. Aime et al.^4^ concluded that olfaction is relevant to food-seeking, and possesses an eco-ethological function in rats; our data are in agreement with their study.

Goetzl and Stone^17^ were the first to discuss the acuity of olfaction and food intake in articles published in 1947^17^ and 1948.^18^ When satiated, the primate orbitofrontal cortex decreases its responsiveness to an odour.^19^ Although olfactory-driven behaviour in humans has not yet been demonstrated in clinical studies, it has been well established in experimental studies. Before the current study, Cameron et al.^1^ were the first to publish a report of olfactory-driven behaviour in humans. They stated that changes in olfactory function can modify feeding behaviour, but the way in which acute negative energy balance impacts olfaction and palatability remains unclear. In their study, 15 subjects (9 males, 6 females) with a mean age of 28.6 ± 4.5 years, a mean initial body weight of 74.7 ± 4.9 kg and a Body Mass Index (BMI) of 25.3 ± 1.4 kg/m^2^, were assessed at baseline (FED) and post-deprivation (FASTED) for nasal chemosensory performance using the “Sniffin’ Sticks” olfactory test. Food palatability ratings were also measured using visual analogue scales. Significant improvements in odour threshold, odour discrimination, and total odour scores (TDI), and higher palatability ratings, were observed during fasting. The authors concluded that fasting for 24 h improves olfactory function; this effect was associated with increased palatability ratings and initial body weight. Further studies are required to confirm the roles of body weight and sex in olfaction and palatability. Similar to Cameron et al.,^1^ we also observed improved olfactory function during fasting, which decreased during satiation. Compared with their study, our results at 16 h were identical to theirs at 24 h, and our group was threefold larger (48 vs. 15).^1^ Recently, Hanci and Altun^20^ conducted another study that included 123 subjects in a prospective design; their results were similar to ours in terms of TDI scores, but there were also differences between the studies. The subjects in Hanci and Altun were scheduled for routine check-ups and fasted for 8 h versus our 16 h fasting period. We suggest that, in the morning, humans exhibit certain physiological changes dependent on the recency of waking, such as increased steroid levels compared to before dinnertime, as per our study. Therefore, our test schedule was optimised compared to that of Hanci and Altun.^20^ Our study is the third concerning smell and fasting in humans, but all three differ in terms of the number of patients included and the fasting durations. Moreover, we found that a fasting threshold of ≤9 h (Group A) affected (i.e., reduced) food intake at dinner to a greater degree, i.e., the satiation period had more effect on individuals with a superior sense of smell (Group A).

There are several limitations to this study. The number of subjects was low and a more-objective method (olfactometry) could have been used; furthermore, we could also have measured the effects of different fasting durations (e.g., 8, 16 and 24 h) in our patient group.

We suggest that, in light of our results pertaining to the medical measurement of olfaction using both olfactometry and sniff tests, evaluations should be performed consistently during periods of either hunger or fullness to achieve more accurate results. Future work could extend our understanding by exploring the relationship between the taste sense and fasting, and by searching for additional hotspots that might improve our knowledge of obesity and associated diseases. This could also aid the discovery of new anti-obesity drugs and therapies.

## Conclusion

As a result, not only do external chemical stimulants affect the olfactory system, but internal chemical and metabolic stimulants are also detected by this system. Increases in olfactory sensitivity during fasting might be related to this pathway, the neurotransmitters and receptors of which should be the subject of further study. Future work should aim to extend this understanding and seek to identify additional hotspots in the brain.

## Conflicts of interest

The authors declare no conflicts of interest.
